# Mal de Debarquement Syndrome: A Case Report

**DOI:** 10.7759/cureus.3270

**Published:** 2018-09-07

**Authors:** Nidhi Shankar Kikkeri, Junaid H Siddiqui

**Affiliations:** 1 Neurology, University of Missouri, Columbia, USA

**Keywords:** cruise trip, disembarkment, imbalance

## Abstract

Mal de Debarquement syndrome (MdDS) is an uncommon neurological disorder seen in women, mostly in their fourth decade of life. It is characterized by a constant sensation of swaying or motion after one disembarks from a vehicle such as a ship or plane following a lengthy trip. These symptoms temporarily subside when the patient is subjected again to passive motion like driving a car. There are no definitive diagnostic tests for Mal de Débarquement syndrome. It is a diagnosis of exclusion and does not have an effective treatment. The symptoms usually resolve spontaneously in about a year. We report a case of a 47-year-old female who presented with a feeling of imbalance following about a four-week cruise, which temporarily subsides during a bicycle ride or a car drive. We report this case, as this condition may not be well-known and probably under-reported. Prospective travelers should be warned and patients can perhaps be cautiously reassured.

## Introduction

The term Mal de Débarquement is derived from a French term that translates to "sickness of disembarkment" [1]. Mal de Débarquement syndrome (MdDS) is a neurological condition, characterized by a continuous perception of self-motion lasting for more than one month from the onset, following disembarkment from a vehicle (ship, cruise, or plane) [2]. This phenomenon was first described by an English physician, Erasmus Darwin, in the year 1796 [3], but it gained more recognition after Brown JJ and Baloh RW published a case series of six patients with a persistent motion-induced subjective balance disorder in the year 1987 [1]. Although MdDS is a diagnosis of exclusion [4] and there are no definitive diagnostic tests available, there are certain features that may suggest this condition. MdDS is usually seen in women in their forties [5] and patients usually describe a constant sensation of rocking, swaying, or bobbing while walking and while lying still [6-7]. Interestingly, these symptoms temporarily subside when patients are re-exposed to passive motion (eg. driving a car) [8-9]. This temporary relief of symptoms is unique to patients with MdDS and hence is used in confirming the diagnosis [10].

## Case presentation

A 47-year-old female patient with a past medical history significant for migraine headaches presented to the neurology clinic for the evaluation of a feeling of imbalance. The symptoms began four months prior to presentation when she returned from a three and a half week cruise. She had been feeling “off-balance” ever since the time she stepped off the cruise. She described the symptom as a feeling of constant motion throughout the day, which persisted while lying down.

To her surprise, she noticed that these symptoms temporarily subsided when she drove a car and even rode a bicycle, but they returned as soon as she got out of the car or off the bicycle. She also reported a history of vertigo about three years ago, which had subsided with positional exercises, and noted that this was a different kind. She denied headaches, nausea, vomiting, tinnitus, or falls.

Prior to her visit to this neurology clinic, the patient was seen by numerous physicians, including an otorhinolaryngologist who had suggested vestibular exercises that did not seem to help her. She was also started on clonazepam, which the patient self-discontinued within a week because it was ineffective.

**Figure 1:**
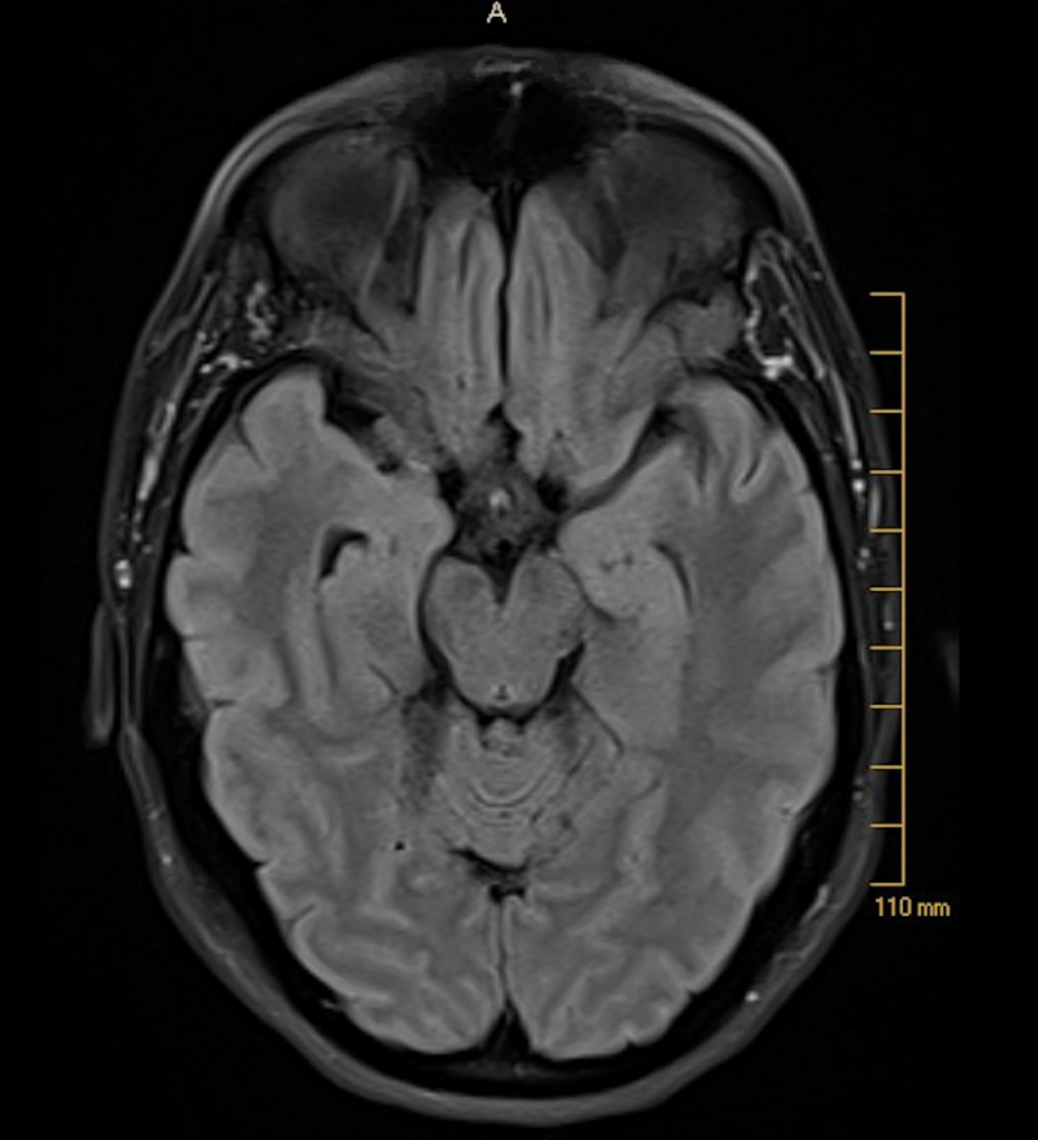
T2/FLAIR image of the axial section of the MRI brain at the level of the Sylvian fissure showing normal findings FLAIR: Fluid-attenuated inversion recovery; MRI: Magnetic resonance imaging.

The physical examination, including a complete neurological examination, was unremarkable. An extensive workup, consisting of magnetic resonance imaging (MRI) brain (Figure 1), video-nystagmography (VNG), oculomotor testing, positional testing, and audiogram were all normal. The patient’s prior laboratory investigations were unremarkable. In this context of a typical history with a normal physical examination and investigations, a provisional diagnosis of Mal de Débarquement syndrome was made after a literature review. The patient was then reassured that her symptoms would hopefully spontaneously resolve in a year.

## Discussion

Mal de Débarquement syndrome (MdDS) is a rare neurological disorder. Only one study has assessed the prevalence of this condition till date and estimated the occurrence to be about 1.3% in a neuro-otologic clinic [2,11]. MdDS usually occurs following prolonged boat or ship travel. As a large number of physicians are unaware of this condition, patients are often misdiagnosed and some patients may never receive the diagnosis. On an average, it is found that MdDS patients visit around 19 physicians before receiving a correct diagnosis [12]. Many patients do not receive the diagnosis for several years, and this can negatively impact their lives.

The pathogenesis of MdDS is not clearly understood. One theory suggests that MdDS is a disorder of neuroplasticity and vestibular adaptation [9]. Genetic predisposition and visual-vestibular conflict are some other theories [1]. Recent research by Cha YH provided some new insight into the biological basis of MdDS using a functional MRI and 18F-fludeoxyglucose positron-emission tomography (18F-FDG–PET) [11]. Patients with persistent MdDS have increased glucose metabolism in the amygdala and the left entorhinal cortex (an area that processes spatial information) and decreased metabolism in the left prefrontal and temporal cortices [11].

The differential diagnoses for MdDS include a vestibular migraine, motion sickness, chronic subjective dizziness, and other otological causes of dizziness [1]. Tests like VNG, MRI brain, computed tomography (CT) scan of the temporal bone, audiogram, and cardiology evaluation can help in differentiating these disorders from MdDS [1]. These tests are normal in patients with MdDS and hence the diagnosis of MdDS is usually clinical. This makes it important for physicians to be aware of this condition to properly diagnose it. Unfortunately, there is no effective treatment for MdDS. However, symptoms resolve spontaneously in most of the patients [1]. Benzodiazepines and anxiolytics are reported to improve the symptoms temporarily [13]. The preferred medication is clonazepam due to its long half-life [1]. A recent study by Cha YH et al. found good tolerability and short-term improvement in symptoms with the use of repetitive transcranial magnetic stimulation [14].

In our case, the patient presented with persistent symptoms after multiple physician visits with normal examinations, lab investigations, and imaging. A history of cruise travel, the nature of symptom onset soon after cessation of motion, and partial symptom relief upon re-exposure to motion activity helped us in diagnosing this condition at her first visit to the neurology clinic.

## Conclusions

Mal de débarquement syndrome is a rare neurological condition that is often unrecognized and misdiagnosed. A thorough clinical history with a high degree of suspicion is needed for recognizing this disorder. Increasing awareness about this disorder among physicians can lead to early diagnosis and prevent multiple physician visits, repeated imaging studies, and potential health care costs. Patients should be reassured about the nature of this disorder and benzodiazepines like clonazepam may be considered.
